# Cemented versus uncemented hemiarthroplasty for displaced femoral neck fractures: A meta-analysis of randomized controlled trails

**DOI:** 10.1097/MD.0000000000014634

**Published:** 2019-02-22

**Authors:** Feng Fei Lin, Yi Fang Chen, Bin Chen, Chao Hui Lin, Ke Zheng

**Affiliations:** a Department of Orthopedic Surgery, Fuzhou Second Hospital Affiliated to Xiamen University, Teaching Hospital of Fujian Medical University; b Fujian University of Traditional Chinese Medicine, Fuzhou, Fujian, China.

**Keywords:** cemented, displaced femoral neck fractures, hemiarthroplasty, meta-analysis, uncemented

## Abstract

**Background:**

The purpose of this meta-analysis was to compare the effectiveness and safety of cemented versus uncemented hemiarthroplasty for displaced femoral neck fractures.

**Methods:**

We searched PUBMED, EMBASE, Cochrane Library, and Google Scholar from their inception to February 2016. All RCTs comparing cemented with uncemented hemiarthroplasty for displaced femoral neck fractures were eligible. The participants who underwent primary hemiarthroplasty for unilateral femoral neck fracture were older than 55 and the mean age of more than 75 years old. For the trials before 2006 used old designed prostheses, so we excluded trails before 2006 which used old designed prostheses. Outcomes of interest include postoperative hip function, Harris hip score (HHS), mortality, reoperation rate, complications, operation time, intraoperative blood loss. Two reviewers independently evaluated the included studies and extracted data into RevMan. Quality Assessments were classified by agreement of 2 authors based on the Cochrane tool.

**Results:**

Seven trials were eligible. Postoperative hip function at 12 months cemented hemiarthroplasty was better than that in uncemented hemiarthroplasty (OR = 0.52, 95% CI, 0.31–0.88; *P* = .01). Postoperative fractures rates in cemented hemiarthroplasty were lower than that in uncemented hemiarthroplasty (OR = 0.09, 95% CI, 0.02–0.38; *P* = .001). Also, the interoperative fracture rates in cemented hemiarthroplasty were lower than that in uncemented hemiarthroplasty (OR = 0.29, 95% CI, 0.13–0.68; *P* = .004). Shorter operation time was achieved in uncemented hemiarthroplasty than that in cemented hemiarthroplasty (WMD = 8.22 min, 95% CI, 5.57–10.86 min; *P*<.00001). There was no significant difference between the 2 groups with HHS, mortality, wound infection, dislocation, general complications, reoperation rate, and intraoperative blood loss.

**Conclusion:**

The available evidence indicates that compared with uncemented hemiarthroplasty cemented hemiarthroplasty achieved better postoperative hip function, less postoperative, and interoperative fractures in displaced femoral neck fracture. Uncemented hemiarthroplasty achieved shorter operation time. There was no difference between the 2 groups with HHS at 1 year, mortality, and complications.

## 1 Introduction

With the increase in the aging population and average life expectancy, hip fractures frequently encountered in patients older than 55 years for osteoporosis.^[1]^

The choice of surgical treatment for displaced femoral neck fractures in adults remains as controversial now. The aim of treatment of a displaced femoral neck fracture in patients is to enable them to walk soon on a stable and painless hip. According to the method of implant fixation, hemiarthroplasty prosthesis can be divided into 2 different types: cemented and uncemented hemiarthroplasty.

The use of uncemented components is known to increase the risk of periprosthetic fracture, and recent data from Rogmark^[2]^ showed a greater risk of reoperations for uncemented hemiarthroplasty in patients with a fracture of the hip. Although cemented fixation has been the standard treatment for patients with a femoral neck fracture, there are reports that the process of cementation increases the risk of cardiopulmonary events.^[3]^ Another disadvantage is that revision hemiarthroplasty will be more difficult in cemented hemiarthroplasty. The question is whether these complications could be avoided by using uncemented fixation.

In the 2010 Cochrane review of arthroplasties^[4]^ (with and without cement) for displaced femoral neck fractures, it was concluded that cemented fixation led to less pain and better mobility, but with no difference in the rate of complications or mortality. However, the authors also reported that they had reviewed mainly old designed prostheses, both cemented and uncemented, and it was a need for more studies that should include modern prostheses.

We performed this meta-analysis with all the randomized controlled studies (RCTs) concerning the comparison of postoperative hip function, postoperative Harris hip score, perioperative mortality, mortality at 1 year, reoperation rate, and complications rates between modern cemented and uncemented techniques for displaced femoral neck fracture in patients from their inception to 2016.

## 2 Methods

### 2.1 Search strategies

A comprehensive search of all studies comparing cemented hemiarthroplasty and uncemented hemiarthroplasty was conducted through the online studies of PUBMED, EMBASE, Cochrane Library, and Google Scholar from their inception to February 2016. The following medical subject headings (MeSH) were searched: femoral neck fracture, hemiarthroplasty, hip fractures, subcapital fractures of the femur, cervical hip fracture, intracapsular proximal femoral fractures. We traced the bibliographies of all retrieved trials and other related publications. We applied no language restriction. For our study is a meta-analysis, the ethical approval was not necessary.

### 2.2 Inclusion criteria and exclusion criteria

All RCTs comparing cemented with uncemented hemiarthroplasty for displaced femoral neck fractures were eligible. The participants were older than 55 and the mean age of more than 75 years old who underwent primary hemiarthroplasty for unilateral femoral neck fracture. Also, if the primary outcome was not postoperative hip function, postoperative Harris hip score, reoperation rate, mortality, and complications rates, then these studies were excluded. Because trials before 2006 were used old designed protheses, the trails before 2006 which used old designed prostheses, for example, they used Bipolar, Thompson and Austin Moore prosthesis were excluded. If there were more than 1 eligible publication from 1 author, the one with higher quality or the most recent publication date would be included. Eligibility issues were among authors, and selected studies underwent a full-text assessment.

### 2.3 Data extraction

Two reviewers independently evaluated the included studies and extracted data into RevMan. Extracted data included publication year and author, mean age and study populations, the type of cemented or uncemented prostheses, garden grade, postoperative hip function, postoperative Harris hip score, residual pain, perioperative mortality, mortality at 1 year, reoperation rate, and complications rates. The complications included intraoperative and postoperative periprosthetic fractures, wound infection, dislocation, cardiovascular and cerebrovascular complications, pulmonary embolism and deep venous thrombosis and general complications such as pneumonia, urinary tract infection, intraoperative blood loss, and operation time. Any disagreement was resolved by discussion with a third reviewer. When there was hemiarthroplasty and total hip arthroplasty in 1 study we just extracted data from hemiarthroplasty. If still more data were required, communication through email would be carried out with the authors.

### 2.4 Outcome measures

The primary outcomes of interest were a postoperative hip function, postoperative Harris hip score, perioperative mortality, mortality at 1 year, reoperation rate, and complications rates. The complications included intraoperative and postoperative periprosthetic fractures, wound infection, dislocation, and general complications such as cardiovascular and cerebrovascular complications, pneumonia, urinary tract infection, etc. The secondary outcomes consisted of intraoperative blood loss and operation time.

### 2.5 Quality assessment

Risk of bias was evaluated by the Cochrane Handbook for Systematic reviews of Interventions guidelines. All studies were classified by agreement of 2 authors as having a low risk of bias, an unclear risk of bias, or a high risk of bias based on the Cochrane tool. This tool takes into account random sequence generation, concealment of the allocation sequence, blinding of participants and personnel, blinding of outcome assessment, incomplete outcome, and selective reporting. Because of the small number of studies (less than 10 studies), no funnel plot was applied.

### 2.6 Statistical analysis

Statistical analysis was performed using Review Manager 5.2. For dichotomous outcomes, weighted averages are reported as relative risk (RR) values or Peto's odds ratio (OR) with associated 95% confidence intervals (CIs). Peto's OR was chosen over RR when the control event rate was exceptionally low (<5%) and the number of subjects randomized in each group of a trial within the given analysis. For continuous outcomes weighted mean differences (WMD) and 95% confidence intervals (CI) were calculated. Statistical heterogeneity was assessed using the I^2^ value≤50% which was considered as no statistical heterogeneity and used the fixed-effects model to estimate the overall summary effect sizes. Otherwise, a random-effects model was used, and a sensitivity analysis would be carried out. *P* value<.05 was considered significant.

## 3 Results

### 3.1 Result of search

**Figure 1:**
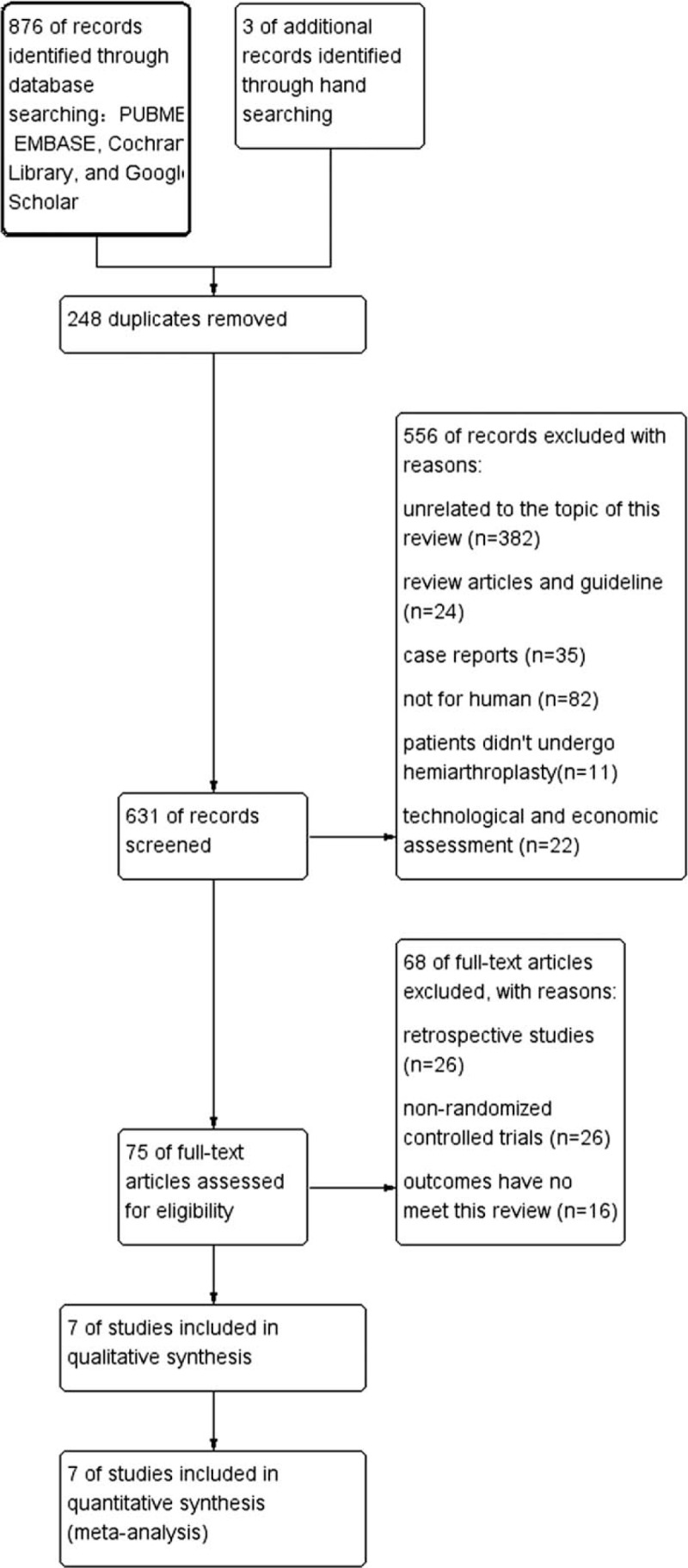
The graph shows a flow diagram of details search and exclusion criteria.

Initially, 879 studies were identified, of which 876 were extracted from electronic databases, and 3 were extracted from reference lists. The search algorithm identified 631 records after deduplication. By screening the titles and abstracts, 556 studies were excluded for unrelated to the topic of this review (n = 382), review and guideline (n = 24), case reports (n = 35), not for human (n = 82), patients did not undergo hemiarthroplasty (n = 11), technological and economic assessment (n = 22). After full-text assessment of 75 articles, 68 studies were excluded for retrospective studies (n = 26), nonrandomized controlled trials (n = 26) and outcomes have no meet this review (n = 16). It resulted in a total of 7 RCTs^[5–11]^ that met the inclusion and exclusion criteria shown in the flow diagram in Fig. 1.

### 3.2 Characteristics of selected studies

We identified 7 randomized controlled trials.^[5–11]^ All selected studies in our meta-analysis were in English and were published from 2009 to 2015. The follow-up period ranged from 1 year to 5 years. The selected study characteristics were summarized in Table 1.

**Table 1:**
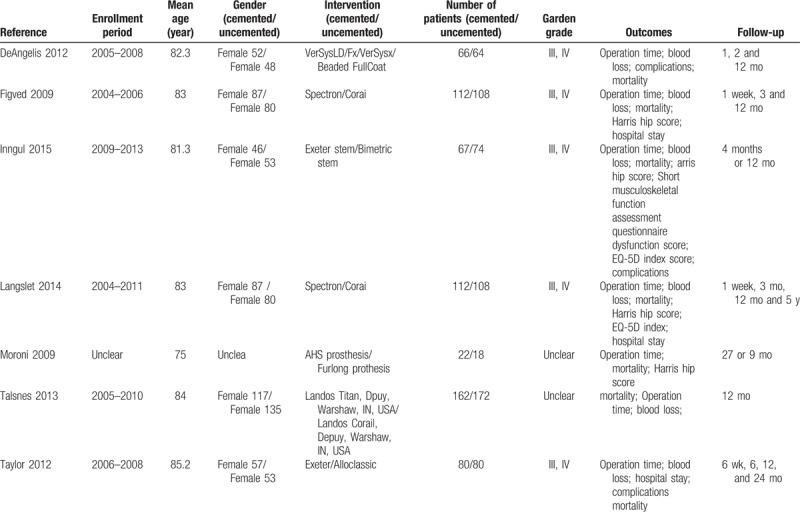
Characteristics of included studies.

### 3.3 Risk of bias

**Figure 2:**
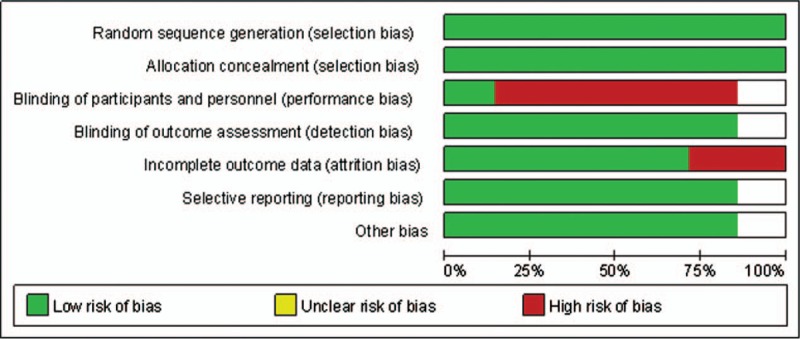
The graph shows the risk of bias graph.

**Figure 3:**
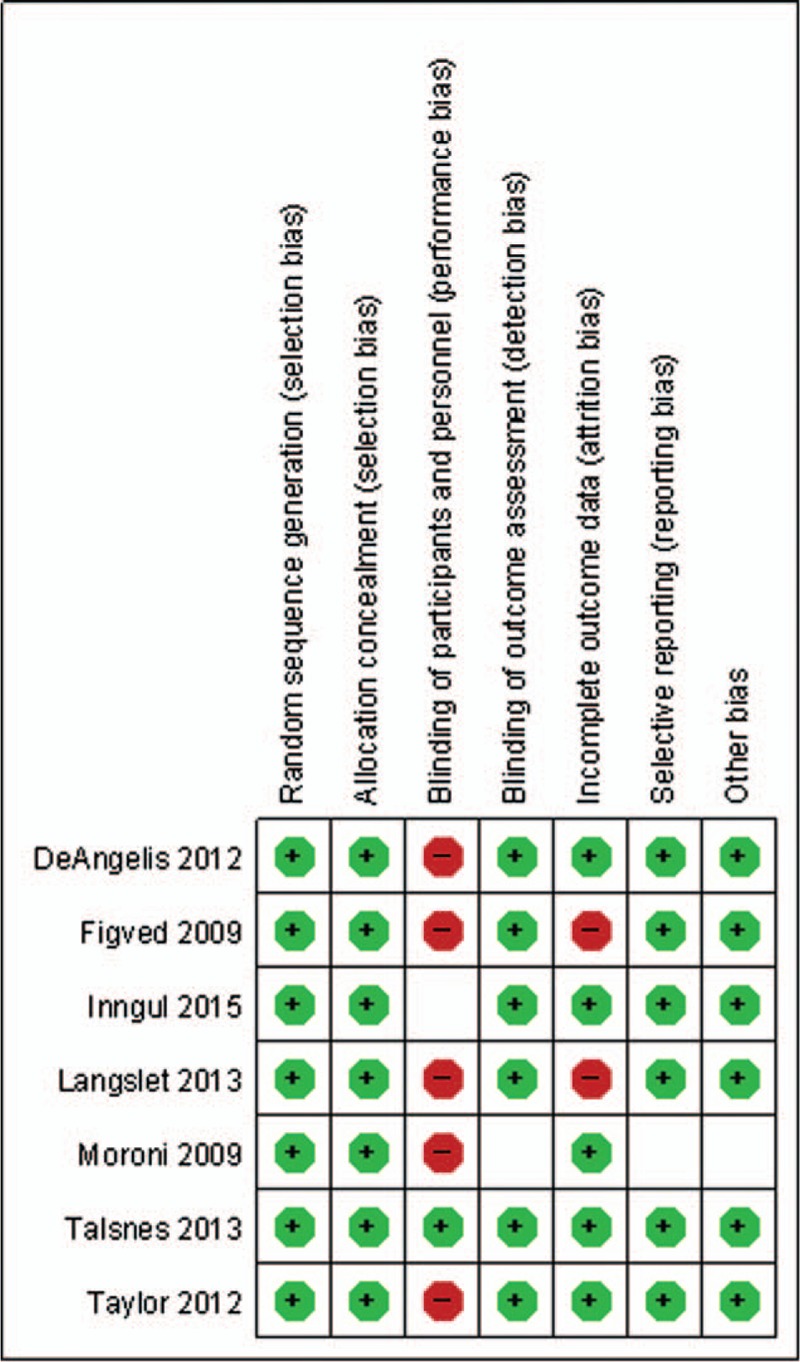
The graph shows the risk of bias summary.

The quality assessment showed that there was no bias in selection, but high bias existed in blinding of participants and personnel because blinding of doctors seemed to be impossible in this study. The detailed risk of bias about methodological quality of the included studies is elaborated and summarized respectively in Figs. 2 and 3.

### 3.4 Meta-analysis results

#### 3.4.1 Postoperative hip function and Harris hip score

**Figure 4:**
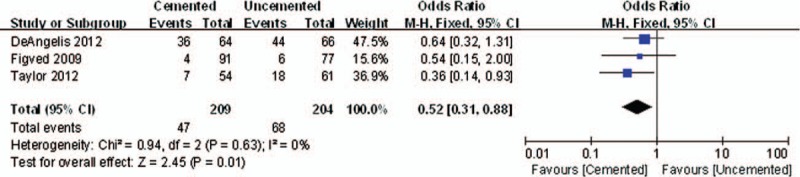
The graph shows a forest plot of relative risk with a confidence interval for postoperative hip function.

For various outcome parameters adopted for the assessment of postoperative hip function, it was difficult to pool all the results. The number of patients requiring assistance adopted by 3 trials was included. At 12 months there was a significant difference between the 2 groups (OR = 0.52, 95% CI, 0.31–0.88; *P* = .01) (Fig. 4). It indicated that postoperative hip function at the 1 year in the cemented group was better than that in the Uncemented group.

**Figure 5:**
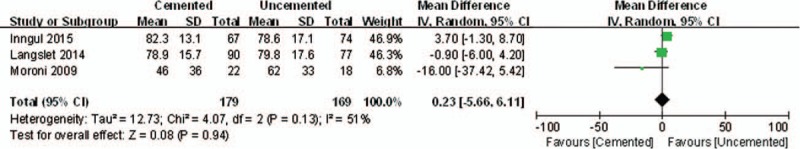
The graph shows a forest plot of relative risk with a confidence interval for HHS.

Three trials were adopted for assessment of postoperative Harris hip score (HHS). For the high heterogeneity, we used the random-effects model. The enrolled studies were just 3, so we could not do a subgroup analysis or sensitivity analysis. There was no significant difference between the 2 groups with HHS at 1 year (MD = 0.23, 95% CI, −5.66–6.11; *P* = .94; random-effects model) (Fig. 5).

### 3.5 Mortality

**Figure 6:**
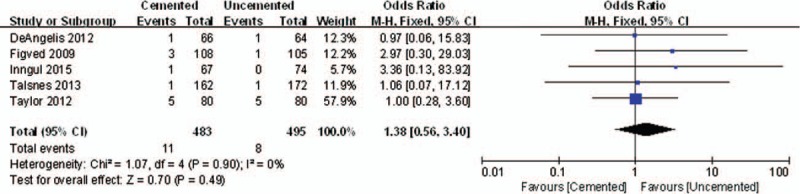
The graph shows a forest plot of relative risk with a confidence interval for perioperative mortality.

**Figure 7:**
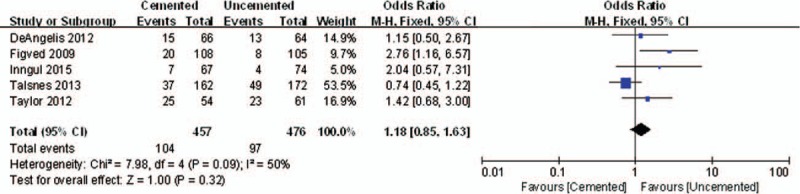
The graph shows a forest plot of relative risk with a confidence interval for postoperative mortality at 1 year.

Five studies reported the results of mortality. There was no significant difference between the 2 groups in perioperative mortality (OR = 1.38, 95% CI, 0.56–3.40; *P* = .49) (Fig. 6). Also, no significant difference was detected between the 2 groups at 1 year for postoperative mortality (OR = 1.18, 95% CI, 0.85–1.63; *P* = .32) (Fig. 7).

### 3.6 Reoperation rate

**Figure 8:**
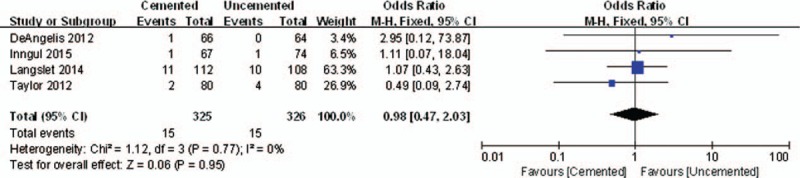
The graph shows a forest plot of relative risk with a confidence interval for the reoperation rate.

All the 4 enrolled trails reported the reoperation rate. The pooled results showed that there was no significant difference between the 2 compared groups in the reoperation rate (OR = 0.98, 95% CI, 0.47–2.03; *P* = .95) (Fig. 8).

### 3.7 Complications

**Figure 9:**
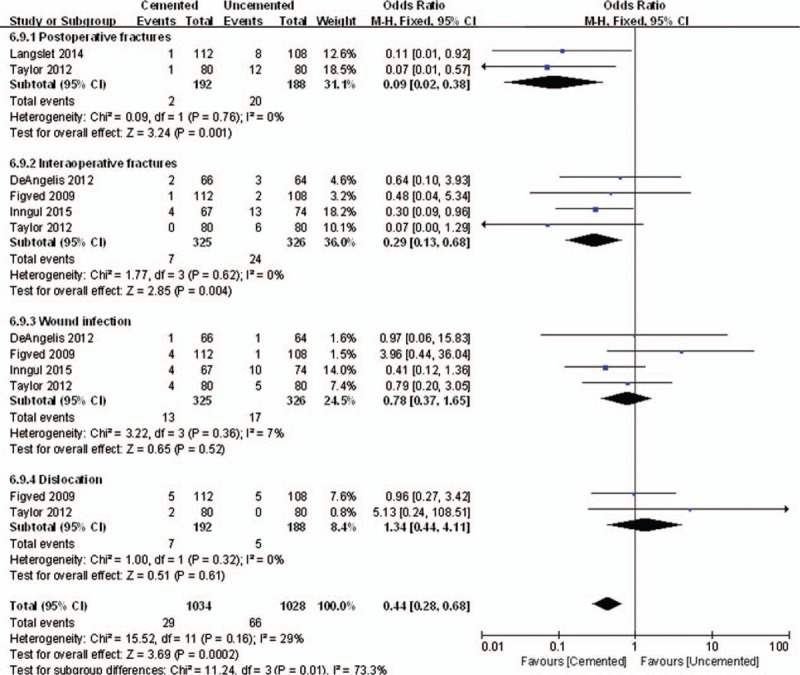
The graph shows a forest plot of relative risk with a confidence interval for local complications.

There was reported complications about postoperative fractures (OR = 0.09, 95% CI, 0.02–0.38; *P* = .001), indicating that postoperative fractures rates in cemented group were lower than that in uncemented group. Also the interoperative fractures rates in cemented group were lower than that in uncemented group (OR = 0.29, 95% CI, 0.13–0.68; *P* = .004). However, there was no significant difference between the 2 groups in wound infection (OR = 0.78, 95% CI, 0.37–1.65; *P* = .52) and dislocation (OR = 1.34, 95% CI, 0.44–4.11; *P* = .61). The summation local complications were less in cemented hemiarthroplasty compared with uncemented hemiarthroplasty (OR = 0.44, 95% CI, 0.28–0.68; *P* = .0002), but the heterogeneity between subgroup differences was high (*P* = .01 *I*^2^ = 73.3%) (Fig. 9).

**Figure 10:**
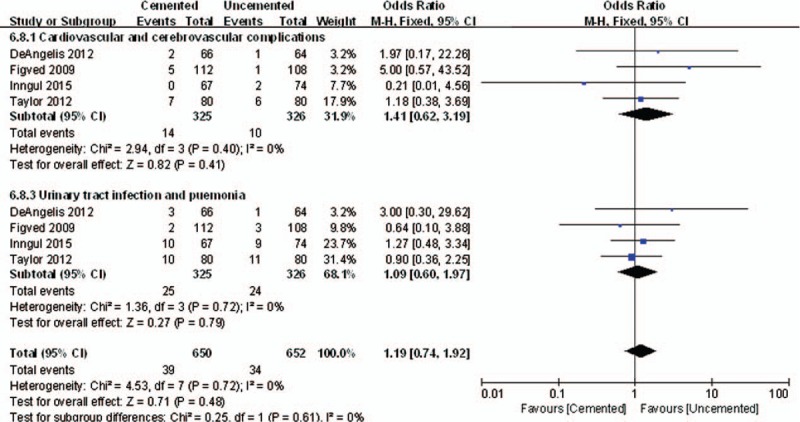
The graph shows a forest plot of relative risk with a confidence interval for general complications.

There were no significant difference between the 2 groups in Cardiovascular and cerebrovascular complications (OR = 1.41, 95% CI, 0.62–3.19; *P* = .41) and urinary tract infection and pneumonia rate (OR = 1.09, 95% CI, 0.60–1.97; *P* = .79). The summation general complications were no significant difference between 2 compared groups (OR = 1.19, 95% CI, 0.74–1.92; *P* = .48 *I*^2^ = 0%) (Fig. 10).

### 3.8 Operation time

**Figure 11:**
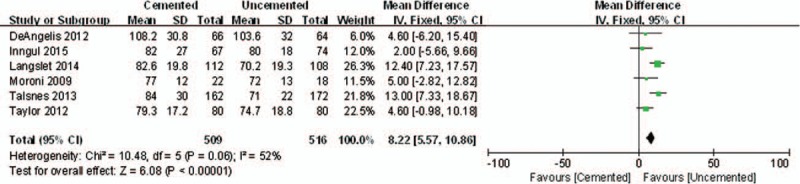
The graph shows a forest plot of relative risk with a confidence interval for operation time.

Six included studies reported operation time. The WMD of operation time was (WMD = 8.22 min, 95% CI, 5.57–10.86 min; *P*<.00001), indicating that the uncemented group achieved a shorter operation time than that in cemented group (Fig. 11).

### 3.9 Interoperative blood loss

**Figure 12:**
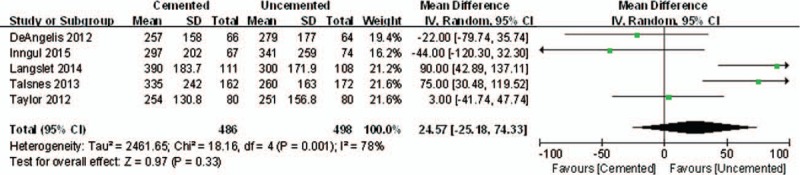
The graph shows a forest plot of relative risk with a confidence interval for interoperative blood loss (random-effects).

This parameter was measured in 5 enrolled studies. Due to the high heterogeneity (*P* = .001, *I*^2^ = 78%), a random-effects model was adopted to pool the data. There was no significant difference between the 2 groups in the intraoperative blood loss (WMD = 24.57 mL, 95% CI, −25.18–74.33 mL; *P* = .33; random-effects model) (Fig. 12).

## 4 Discussion

In our study, it indicated a better postoperative hip function in cemented prostheses at 1 year. The fractures rates were more frequent in uncemented hemiarthroplasty than in cemented hemiarthroplasty. We were also able to find that the increased operation time associated with cement but did not increase intraoperative blood loss. The summation local complications were less in cemented hemiarthroplasty compared with uncemented hemiarthroplasty. However, there was also not able to show any significant difference in the rates of general complications.

We enrolled the number of patients requiring assistance with ambulation from each trial, which was the common parameter in assessing the postoperative hip function. It indicated a better postoperative hip function in cemented prostheses at 1 year. In Li T's meta-analysis, they also found a better postoperative hip function in cemented prostheses at 1 year. But in their meta-analysis, they included some old designed prostheses.^[12]^ Also, we used HHS to assessed postoperative hip function, but there were just 3 modern studies enrolled. We can see that HHS was similar between the cemented group and the uncemented group. It needs further researches with large samples and standardized hip function scoring systems to confirm these findings and elucidate the potential advantages of cemented techniques in postoperative hip function recovery.

There just 2 studies reported residual pain. So we could not pool them up. It showed that the cemented group had less residual pain compared with the uncemented group in every single study. According to Langslet's report, better pain relief was achieved in uncemented hydroxylapatite coated implant. In this 2 studies, some enrolled trials adopted hydroxylapatite coated implant while other trials used nonhydroxylapatite coated prostheses. It was indicated that the cemented group was associated with less residual pain. Luo et al^[13]^had also demonstrated this conclusion in their meta-analysis.

Interoperative and postoperative fractures owing to the failure of osseointegration are risk factors when using uncemented stems. There is evidence that coating titanium stems with hydroxyapatite improves bone ingrowth. However, our review included some current studies comparing modern hydroxyapatite coated uncemented hemiarthroplasty with cemented hemiarthroplasty. There was significant difference in the rates of interoperative and postoperative fractures between 2 groups. The fractures rates were more frequent in uncemented hemiarthroplasty than in cemented hemiarthroplasty. That is why we use cemented hemiarthroplasty more often in osteoporosis patients. But we found no significant difference in wound infection and dislocation between the 2 groups.

The theoretical advantages of using an uncemented prosthesis are to avoid immediate fatal complications related to the cement implantation syndrome.^[3]^ It was found that there was an increased rate of mortality on the first postoperative day for cemented implants and an increased rate of mortality for patients with uncemented implants after 1 year following surgery. A retrospective study comparing cemented hemiarthroplasty with uncemented hemiarthroplasty showed that uncemented implants were related to significantly higher rates of myocardial infarction and lower respiratory tract infection within 30 days compared with cemented implants.^[14]^ Interestingly, our study was not able to show any significant difference in the rates of perioperative mortality and postoperative mortality at 1 year. Some studies suggest avoiding the use of cement for the occurrence of pulmonary embolism. The mechanism attributed to direct cement toxicity and embolism of bone marrow contests. But in our meta-analysis, just 2 studies recorded the occurrence of pulmonary embolism so we cannot pool them up. But there was not able to show any significant difference in the rates of pulmonary embolism in every single study.

There was also not able to show any significant difference in the rates of urinary tract infection, cardiovascular and cerebrovascular complications between the 2 groups.

In this meta-analysis, we were able to demonstrate that the increased operation time associated with cement but did not increase intraoperative blood loss. The prolonged operation time may result from the process of cement insertion and the waiting time for solidification of cement. The pooled results for intraoperative blood loss with high heterogeneity (*P* = .003, *I*^2^ = 73%) showed that there was no significant difference between the 2 groups. Some studies adopted a hydroxylapatite-coated implant while other trials used non-hydroxylapatite-coated prostheses, which was regarded as the source of heterogeneity. So we used the random-effects model. Ning et al^[15]^ had also demonstrated this conclusion in their meta-analysis. Also, they included some old designed prostheses.

One of the limitations of this meta-analysis was that as surgical intervention, it was not possible to blind the surgical or anesthesia staff. The second was that just 1 included study followed up to 5 years, most of the included studies followed up just for 1 year. We look forward to seeing more RCT can follow up to 5 years so that we can analyze the long-term function of the postoperative hip. The third was that the pooled-analysis of postoperative hip function and Harris hip score just included 3 studies. For there were not so many studies that recorded Harris hip score, we also look forward to seeing more RCT study about Harris hip score.

Conclusions: The available evidence indicates that compared with uncemented hemiarthroplasty cemented hemiarthroplasty achieves better postoperative hip function, less postoperative and interoperative fractures. Uncemented hemiarthroplasty achieves shorter operation time. So osteoporosis patients have more often used cemented hemiarthroplasty.

## Author contributions

**Conceptualization:** Feng Fei Lin, Yi Fan Chen, Bin Chen, Ke Zheng.

**Data curation:** Feng Fei Lin, Yi Fan Chen, Chao Hui Lin, Ke Zheng.

**Formal analysis:** Feng Fei Lin, Yi Fan Chen, Bin chen, Chao Hui Lin, Ke Zheng.

**Investigation:** Feng Fei Lin, Yi Fan Chen, Bin chen, Ke Zheng.

**Methodology:** Feng Fei Lin, Yi Fan Chen, Bin chen, Chao Hui Lin.

**Project administration:** Feng Fei Lin, Bin chen, Chao Hui Lin, Ke Zheng.

**Resources:** Feng Fei Lin, Bin chen.

**Software:** Yi Fan Chen, Bin chen, Chao Hui Lin.

**Supervision:** Feng Fei Lin, Ke Zheng.

**Validation:** Feng Fei Lin, Yi Fan Chen.

**Visualization:** Feng Fei Lin, Chao Hui Lin.

**Writing – original draft:** Feng Fei Lin, Chao Hui Lin, Ke Zheng.

**Writing – review & editing:** Feng Fei Lin, Ke Zheng.

Feng Fei Lin orcid: 0000-0002-4360-0386.
