# Cemented versus uncemented hemiarthroplasty for displaced femoral neck fractures: A meta-analysis of randomized controlled trails: Erratum

**DOI:** 10.1097/MD.0000000000015216

**Published:** 2019-04-05

In the article, “Cemented versus uncemented hemiarthroplasty for displaced femoral neck fractures: A meta-analysis of randomized controlled trails”,^[1]^ which appeared in Volume 98, Issue 8 of *Medicine*, Dr. Yi Fan Chen's name appears incorrectly as Yi Fang Chen.
